# Analytical Prediction of Subsurface Damages and Surface Quality in Vibration-Assisted Polishing Process of Silicon Carbide Ceramics

**DOI:** 10.3390/ma12101690

**Published:** 2019-05-24

**Authors:** Yan Gu, Yan Zhou, Jieqiong Lin, Allen Yi, Mingshuo Kang, Hao Lu, Zisu Xu

**Affiliations:** 1 Key Laboratory of Micro/Nano and Ultra-precision Manufacturing, School of Mechatronic Engineering; Changchun University of Technology, Changchun 130012, China; zhouyan0920@163.com (Y.Z.); kmsdeyouxiang123@163.com (M.K.); nim578114@163.com (H.L.); 15568607008@163.com (Z.X.); 2 Department of Industrial, Welding and Systems Engineering, Ohio State University, Columbus, OH 43210, USA; yi.71@osu.edu

**Keywords:** SiC ceramics, prediction of subsurface damages, vibration-assisted polishing, finite element simulation

## Abstract

Subsurface damages and surface roughness are two significant parameters which determine the performance of silicon carbide (SiC) ceramics. Subsurface damages (SSD) induced by conventional polishing could seriously affect the service life of the workpiece. To address this problem, vibration-assisted polishing (VAP) was developed to machine hard and brittle materials, because the vibration-assisted machine (VAM) can increase the critical cutting depth to improve the surface integrity of materials. In this paper, a two-dimensional (2D) VAM system is used to polish SiC ceramics. Moreover, a theoretical SSD model is constructed to predict the SSD. Furthermore, finite element simulation (FEM) is adopted to analyze the effects of different VAP parameters on SSD. Finally, a series of scratches and VAP experiments are conducted on the independent precision polishing machine to investigate the effects of polishing parameters on brittle–ductile transition and SSD.

## 1. Introduction

With the rapid development of space optics, the dimensional stability of optical components is widely applied in the aerospace industry. Silicon carbide (SiC) ceramics are a representative material due to its inherent features, for example, low density, low thermal coefficient, stable chemical properties, and high-temperature wear resistance. In general, the machining process of SiC mainly consists of wire sawing, grinding, lapping, and polishing. Among the various machining methods, polishing is the last step to obtain good surface integrity. However, SiC ceramics have poor machinability due to their high hardness and brittleness [1,2,3,4]. The surface and subsurface damages may seriously deteriorate the quality of optical parts under thermal load during machining [5]. Such unsatisfactory surface integrity and high cost limit the application of SiC ceramics. Therefore, many scholars conducted work to determine appropriate methods to improve this problem.

In conventional precision polishing, abrasive polishing is the most commonly adopted method. In order to obtain better surface integrity, some researchers recently developed an effective method to prevent surface and subsurface damages on SiC ceramics, which is called vibration-assisted polishing (VAP) [6,7]. It is also considered to be the preferred method for machining hard and brittle materials, as it increases the critical cut depth and reduces the cutting force [8,9,10]. A number of studies focused on utilizing one-dimensional (1D), two-dimensional (2D), or three-dimensional (3D) evibration systems to machine materials [11,12,13]. Due to the complexity of 3D vibration system design, 1D and 2D vibration systems are appropriate choices for some researchers, especially the latter, which recently become a promising method for processing hard and brittle materials. Gou et al. used different vibrating systems to machine tungsten carbide, and the results showed that, compared with 1D vibrating systems, 2D vibrating systems could generate better surface roughness with a higher material removal rate [14]. Therefore, 2D vibration-assisted polishing was developed to polish SiC ceramics with a surface roughness of 35 nm. These studies indicated that the vibration-assisted machine (VAM) is an effective machine method to suppress subsurface damage and achieve lower surface roughness during hard and brittle material processing.

At present, the Preston equation (dh=kpvdt) is applied in the investigation of polishing mechanisms [15,16]. However, the constant k does not accurately describe the relationship between abrasive particles and material removal. Therefore, some studies focused on the complex motion process of abrasives and workpiece surface during the polishing process [17,18]. Earlier, Law et al. found the median cracks arising from subsurface flaws and built a model for crack initiation [19]. In this way, the interactions between the workpiece and the abrasive grains, as well as the formation of subsurface damages (SSD), could be clearly understood.

To investigate the removal mechanism for hard and brittle materials, a lot of research was carried out using mathematical models, finite element modeling (FEM), and experiments. Aida et al. investigated the material removal characteristics of GaN, including the relationship between the SSD depth and the abrasive grain size [20]. The results showed that GaN was similar to other brittle materials, and the SSD depth was decreased by using a relatively smaller abrasive size. Li et al. developed a mathematical model based on the Lambropoulos model to calculate SSD depth, which can also rapidly and accurately predict surface roughness [21]. Zhang et al. established an SSD prediction model considering grinding parameters and spindle dynamics [22]. Esmaeilzare et al. proposed a prediction model to investigate the influences of different process parameters on the surface roughness and SSD depth of Zerodur® ceramics based on statistical analysis [23]. Furthermore, Xiao et al. studied theoretical formulas to calculate the roughness and SSD depth of fused silica [24].

At present, the establishment of SSD models based on indentation fracture mechanics is considered as an effective method to predict SSD depth. However, due to the inaccuracy of the mathematical model, many other scholars attempted to achieve a more accurate SSD depth through experimental exploration. Lucca et al. presented a variety of techniques to evaluate the surface alterations of some ceramics and glasses during processing [25]. Ding et al. investigated the surface damages and SSD under different grinding parameters through a series of single-factor experiments [26]. Compared with conventional grinding, the surface and subsurface damages were improved through introduced ultrasonic vibration. Different grinding parameters could change grinding force into a relatively lower force, which is better for surface/subsurface quality. Moreover, Jiang et al. reported experimental investigations of the brittle material removal fraction to assess the relationship between surface roughness and SSD depth of optical glass during the grinding of brittle materials. Meanwhile, a series of experiments were designed to explore the SSD depth value with different grinding parameters [27,28].

However, the experimental testing occupies massive time and expends high cost; thus, FEM is an effective means to analysis and test values of fracture for multiple materials such as metal alloys and ceramic materials [29]. Cervino et al. investigated how dental implant material was held against the masticatory strength under different directions of dynamic load [30]. The data of this virtual model showed all the features of different prosthetic retention systems under the masticatory load. Based on the advantages of FEM’s wide applicability, some FEM software (e.g. ABQUESE and Analysis) is also widely used to predict subsurface damages of different materials. For example, Komandur et al. used molecular dynamics simulations to study the relationships between cutting results and a combination of the tool edge radius and the cut depth [31]. The change of cutting force during the cutting process, including thrust, force ratio, specific energy, and subsurface deformation, was studied in detail, which was significantly affected by the combination of tool geometry and cut depth. Based on the virtual abrasives with a truncated polyhedral shape, Wan et al. simulated the SSD depth of silicon nitride [32]. The results of the simulation matched well with the experimental results. Based on the Johnson–Holmquist model, Pashmforoush reported a numerical simulation of 2D single grit, which was conducted to analyze the SSD depth of the grinding process using ABAQUS/Explicit [33]. Based on previously published literature, we studied the SSD of SiC in conventional polishing. However, conventional polishing is rarely able to achieve a reduction in SSD [34]. Therefore, a novel processing method, VAP, was used to improve processing quality and inhibit SSD of hard and brittle materials. It is also important for SSD inhibition to investigate the effect of processing parameters.

Motivated by the abovementioned literature, a theoretical model of SSD was established to evaluate crack length, which was based on the removal mechanism for VAP SiC ceramics in this paper. Then, in order to explore the relationship between the SSD and processing parameters, a 3D FEM model was applied to simulate single-abrasive VAP SiC ceramics with various processing parameters (vertical amplitude, lateral amplitude, frequency, and abrasive grain size). At the same time, a series of scratch tests and single-factor experiments were conducted to validate the reliability of the VAP and theoretical model of SSD.

## 2. Theoretical analysis

### 2.1. VAP system

**Figure 1:**
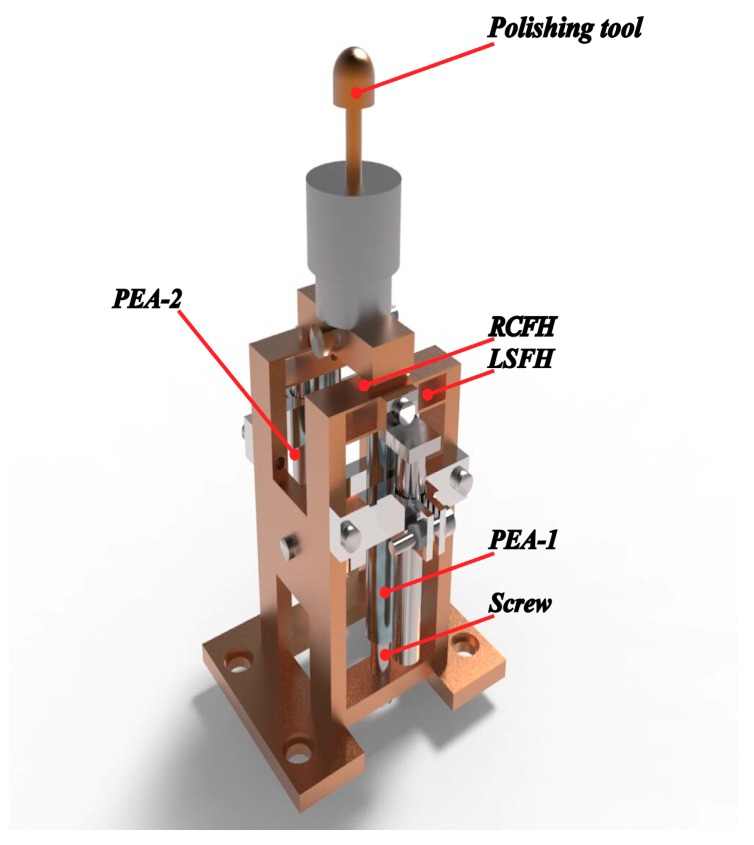
Mechanical structure of the vibration-assisted polishing (VAP) system.

**Figure 2:**
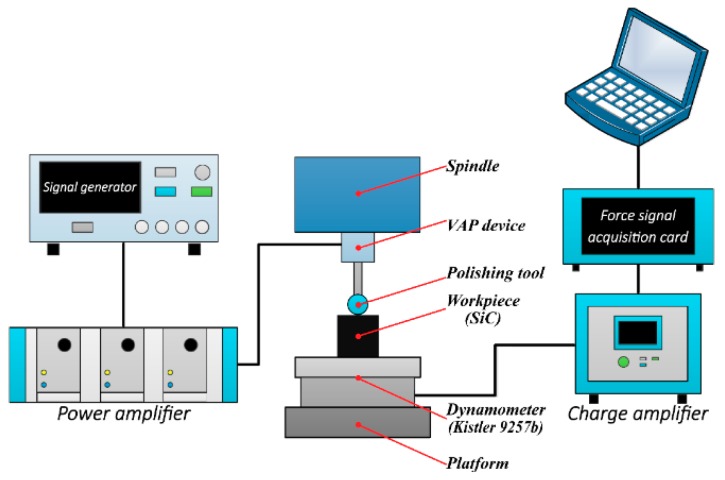
The process of VAP.

The mechanical structure of the VAP system based on flexure hinges is shown in Figure 1 [35]. Leaf spring flexure hinges (LSFHs) and right circular flexure hinges (RCFHs) play an important role in guiding the motion of the VAP system. The VAP device can be driven by two piezoelectric actuators (PEAs)—PEA-1 and PEA-2. In order to ensure the pre-tightening force of the flexure-based device, the screw pre-tightening method was adopted. The schematic diagram of the VAP system is shown in Figure 2.

### 2.2. Kinematical analysis

Deformation not only occurs on the LSFHs, but also on the RCFHs. Because the tool holder can be simplified as a rigid bar, it is appropriate to make an assumption that the motion is generated by flexure hinges.

**Figure 3:**
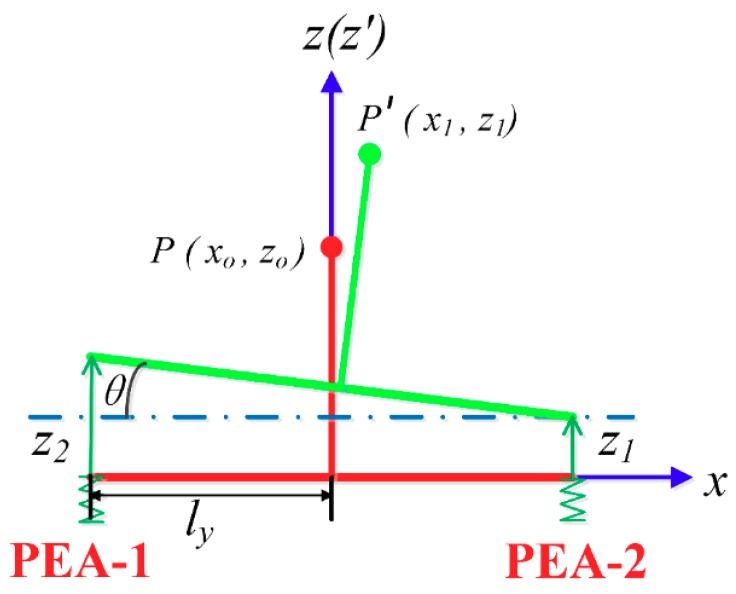
Kinematical schematic.

As shown in Figure 3, the position of the tool tip can be denoted as $P\left(x_{0},z_{0}\right)$ with input displacement of the PEAs set to zero in the coordinate system O−xyz. $P^{\prime }\left(x_{1},z_{1}\right)$ is the instantaneous position of the polishing tip when the PEA-1 outputs a displacement $z_{1}$ and PEA-2 outputs a displacement $z_{2}$, while $l_{y}$ is half the distance of the end beam length.

In term of Figure 3, $P^{\prime }\left(x_{1},z_{1}\right)$ and θ can be obtained as follows:

(1) $$\left\{ \begin{array}{l} x_{1}=z_{0}\sin\theta \\ z_{1}=\frac{z_{1}+z_{2}}{2}+z_{0}\cos\theta \\ \theta =\tan^{-1}\left(\frac{z_{2}-z_{1}}{2l_{y}}\right) \end{array}\right..$$

In the experiment, when two sinusoidal voltages are input to the PEAs, the moving displacement of the tool tip with respect to the PEAs can be expressed as

(2) $$\left\{ \begin{array}{l} z_{1}=A_{1}\sin\left(2\pi ft\right)\\ z_{2}=A_{2}\sin\left(2\pi ft+\varphi \right) \end{array}\right.,$$

where f is the signal frequency, t is the time, φ is the phase difference of the input signals, and $A_{1}$ and $A_{2}$ are the two PEA output displacements along the *Z*-axis.

In order to simplify the equation, it is assumed that $\varphi =\frac{\pi }{2}$; therefore, the single abrasive grain motion of the tool tip can be expressed as

(3) $$\left\{ \begin{array}{l} x=z_{0}\frac{A_{2}\cos(2\pi ft)-A_{1}\sin(2\pi ft)}{\sqrt{4l_{y}{}^{2}+{\left[A_{2}\cos(2\pi ft)-A_{1}\sin(2\pi ft)\right]}^{2}}}+vt\\ z=\frac{\sin(2\pi ft)+\cos\left(2\pi ft\right)}{2}+z_{0}\frac{2l_{y}}{\sqrt{4l_{y}{}^{2}+\left[A_{2}\cos(2\pi ft)-A_{1}\sin(2\pi ft)\right]}} \end{array}\right..$$

Based on the first derivative of the tool motion displacement versus time, the single abrasive velocity with respect to the workpiece can be obtained as follows:

(4) $$\left\{ \begin{array}{l} v_{x}=x^{\prime }\left(t\right)=\frac{dx}{dt}\\ v_{z}=z^{\prime }\left(t\right)=\frac{dz}{dt} \end{array}\right..$$

**Figure 4:**
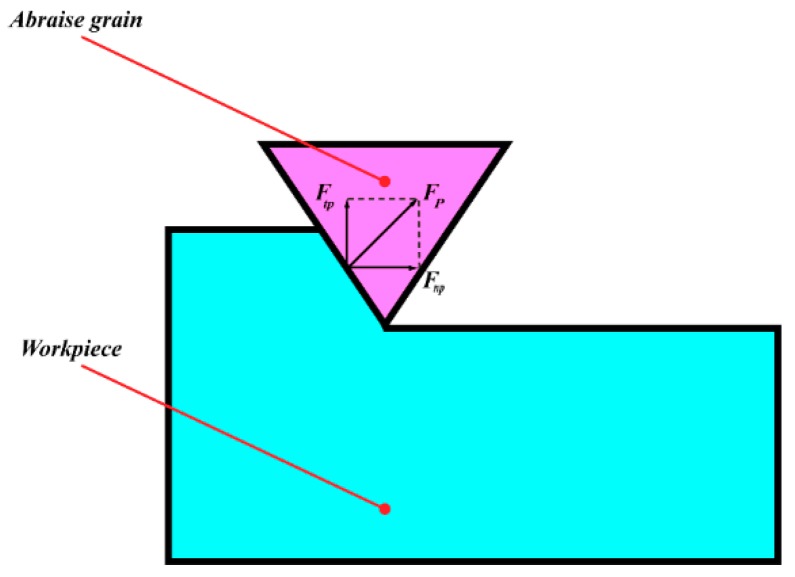
Force model for single abrasive processing.

The polishing is actually a complex process that is not quantitatively removed. To explore this process, the polishing is usually simplified to the scratch process of a signal abrasive, as shown in Figure 4. For conventional polishing, the polishing force constitutes pressure and friction forces between the abrasives and the workpiece. The pressure force of VAP can be expressed by the pulse force. Therefore, the normal and tangential forces of a single grain can be determined as

(5) $$\left\{ \begin{array}{l} F_{t}=F_{tp}+F_{tf}\\ F_{n}=F_{np}+F_{nf} \end{array}\right.$$

where $F_{tp}$ and $F_{nf}$ are the tangential and normal of the pulse force, respectively, $F_{np}$ and $F_{nf}$ are the tangential and normal of the friction force, and $F_{t}$ and $F_{n}$ are the tangential and normal of the polishing force.

The tangential force of the single abrasive can be expressed as

(6) $$F_{tp}=\sqrt{F_{tx}{}^{2}+F_{tz}{}^{2}},$$

where $F_{tx}$ and $F_{tz}$ are the pulse force along the x- and y-directions, respectively.

The equivalent mass of the abrasive is M. According to the impulse theorem, the pulse force acting on the abrasive can be derived as

(7) $$\left\{ \begin{array}{l} F_{tx}\left(t\right)=M\cdot \frac{x^{\prime }\left(t\right)}{\Delta t}\\ F_{tz}\left(t\right)=M\cdot \frac{z^{\prime }\left(t\right)}{\Delta t} \end{array}\right..$$

Then, in the 2D vibration, the normal force of the grain is

(8) $$F_{tp}=\sqrt{F_{tx}{}^{2}+F_{tz}{}^{2}}\tan\alpha ,$$

where the abrasives are considered to be tapered, and α is the half apex angle. 

The tangential and normal friction force of abrasive is

(9) $$\left\{ \begin{array}{l} F_{tf}=\mu F_{{}_{np}}\\ F_{nf}=\mu F_{{}_{tp}} \end{array}\right.,$$

where μ is the coefficient of friction between workpiece and abrasives.

Therefore, the VAP force of the single abrasive can be determined as

(10) $$F=\sqrt{F_{t}^{2}+F_{n}^{2}}.$$

### 2.3. SSD Models

**Figure 5:**
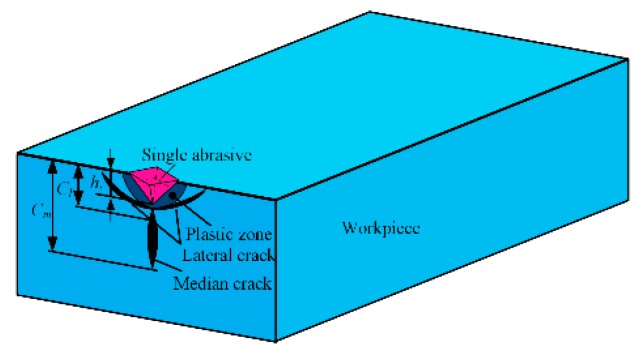
Scratch process of single abrasive grain

**Figure 6:**
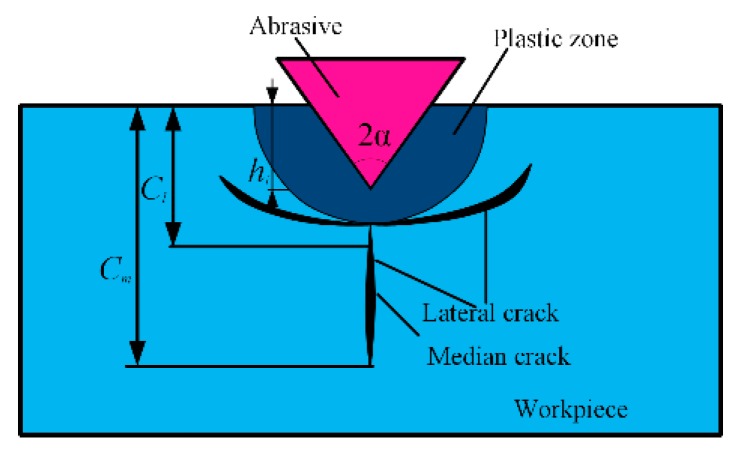
Subsurface damage (SSD) model.

The polishing process can be simplified as a single abrasive scratch on the workpiece, as shown in Figure 5. A novel polishing system combines the simple characteristic of the vibration-assisted flexure-based device and a computer numerical control polishing machine. The abrasive grain is pressed into the workpiece, which leads to median cracks distributed below the polishing surface, as shown in Figure 6. The symbol $c_{m}$ is the length of the median cracks, $c_{l}$ is the depth from the bottom of the lateral crack to the plastic zone, $h_{i}$ is the abrasive penetrating depth with the VAP load, and α is the half apex angle of the abrasive.

There are two forms of cracks, including lateral cracks and median cracks, during the VAP of hard and brittle materials, which cause SSD and degradation of the material strength [36]. 

The median crack extends perpendicularly to the processing surface with an incisive abrasive, which can be classified as an SSD. The depth of the median cracks is generally considered to be the SSD depth. The depth of the median cracks *c_m_* can be considered as a parameter to evaluate the functional properties of the materials [37].

(11) cm=0.206(E⋅Hs)13(Kc⋅β)23(cotα)49(tanα)43⋅(hi)43,

where $H_{s}$ is the scratch hardness, $K_{c}$ is the fracture toughness, and β is a constant that is determined by the elastic recovery of the materials. According to the material properties of SiC, $K_{c}$ is 3.5 MPa⋅m(1/2) and β=0.363.

The calculation of SSD can be expressed by Equation (12) based on the relationship between the abrasives and the SiC.

(12) $$SSD=\max\left(c_{m}\right).$$

The plastic deformation force of the single abrasive intruding into the workpiece at a depth of $h_{i}$ can be obtained from Equation (13) based on the theory of elastic mechanics [38].

(13) $$h_{i}=\sqrt{\frac{F}{2H_{w}}}$$

Combining Equations (11)–(13), the SSD can be obtained as follows:

(14) $$SSD=\max\left[\lambda {\left(\frac{F}{2H_{w}}\right)}^{2/3}\right],$$

where λ=0.206(E⋅Hs)13(Kc⋅β)23(cotα)49(tanα)43.

The material parameters of SiC ceramics are shown in Table 1.

## 3. Simulation analysis and results

### 3.1. Constitutive Models

Considering that the removal mechanism in SiC processing mainly aims at the prevention of brittle fractures, the Johnson–Holmquist (JH-2) model can be applied, because it is based on elastic–plastic damage, which can be used to simulate the mechanical behaviors of brittle materials under high strain, high strain rate, and large pressures [39]. The JH-2 model can be described by Equation (15).

(15) $$\sigma =\left(1+C\ln\overset{\cdot }{\varepsilon }\right)\sigma_{HEL}\left[A{\left(\frac{P+T}{P_{HEL}}\right)}^{N}-D\left(A{\left(\frac{P+T}{P_{HEL}}\right)}^{N}-B{\left(\frac{P}{P_{HEL}}\right)}^{M}\right)\right],$$

where σ is the equivalent stress of material under the hydrostatic pressure P and the strain rate $\overset{\cdot }{\varepsilon }$, D is the material damage coefficient, and T (GPa) is the maximum hydrostatic tensile strength. $\sigma_{HEL}$ is the equivalent stress at the Hugoniot elastic limit (HEL). $P_{HEL}$ is the pressure at the HEL. A, B, C, M, and N are predetermined parameters based on SiC ceramic properties. 

### 3.2. Simulation Methods

**Figure 7:**
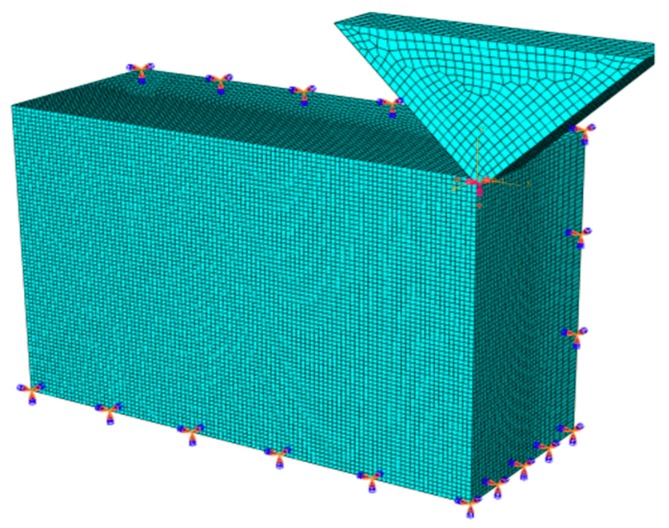
The geometric structure and boundary condition of the three-dimensional (3D) finite element model (FEM).

In order to qualitatively analyze the diversified evolutions of SSD during the SiC VAP process, a 3D single abrasive model was established using ABAQUS/Explicit. The model size was set as 18 μm × 12 μm × 7 μm, as shown in Figure 7. The meshing model used the simplified eight-node hexahedron integral element C3D8R (C represents a physical unit. 3D represents three-dimensional and 8 represents eight-node). The boundary conditions of the workpiece were set as bottom and sides which were fully constrained, and a penalty method was used to simulate the contact between the abrasive and the workpiece. In order to achieve the vibration of a single abrasive, two assignment curves were set in the boundary condition module. The vibration of the abrasive was adjusted by inputting different frequencies, vertical amplitudes, and lateral amplitudes. In order to reduce the simulation time and improve efficiency of the simulation, the mesh of the contact area on the workpiece and the abrasive particles should undergo densification. The sparse mesh can be applied to other parts. Accurate results could be obtained based on the densification mesh, and a sparse mesh can reduce calculation time. For VAP SiC ceramics, the tangential and normal frictions were generated between the abrasive grains and the workpiece, the coefficient of which cannot be ignored. Here, such a friction coefficient was set to 0.3. More time- and cost-effective calculations were achieved using the mass scaling method, in which the scaling factor was set as 30. To simplify the simulation process, the temperature effect of the polishing liquid, the multi-abrasive coupling problem, and tool wear were not considered. The abrasive was served as rigid, and the constitutive material model for the SiC workpiece was defined based on the JH-2 damage model with the parameters listed in Table 2 [40]. The VAP simulation parameters are shown in Table 3.

### 3.3. Simulation and Discussion

#### 3.3.1. VAP process of a single abrasive

**Figure 8:**
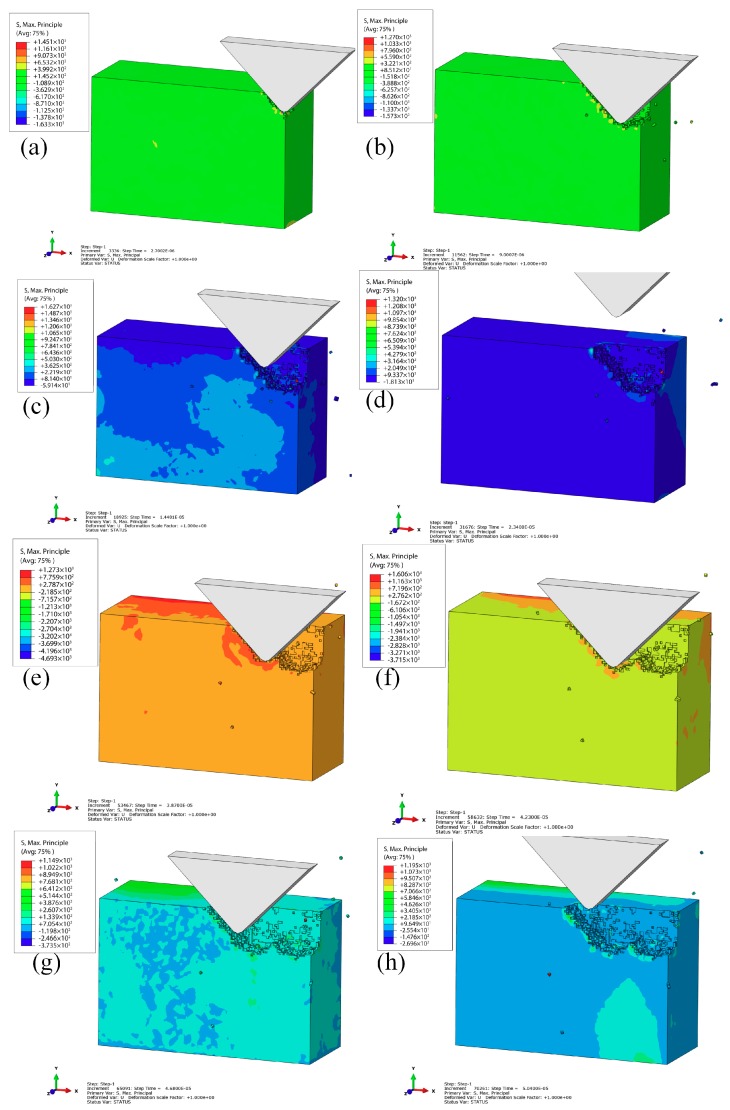
Brittle removal process of the silicon carbide (SiC) VAP: (**a**) t = 2.7 × 10^−6^ s; (**b**) t = 9 × 10^−6^ s; (**c**) t = 1.44 × 10^−5^ s; (**d**) t = 2.34 × 10^−5^ s; (**e**) t = 3.87 × 10^−5^ s; (**f**) t = 4.23 × 10^−5^ s; (**g**) t = 4.68 × 10^−5^ s; (**h**) t = 5.04 × 10^−5^ s.

Figure 8a–f show the removal process of VAP SiC ceramics, where vertical amplitude was 2 μm, the lateral amplitude was 3 μm, vibration frequency was 30 kHz, the apex angle of abrasive was $\alpha =90^{{}^{\circ}}$, and the feed speed was 100 mm/s. In the initial stage, the abrasive grain contacted with the workpiece under the action of VAP. At this time, the principal stress was concentrated at the front and bottom of the abrasive; meanwhile, the nominal depth of cut increased, and some tiny cracks began to occur, as shown in Figure 8a,b. Then, with the movement of the abrasive along the tool path (Figure 8c,d), the increase of stress led to the tiny crack propagation, and the abrasive grain was temporarily separated from the processed surface. In the meantime, the single motion cycle of the abrasive was over. Furthermore, the abrasive initiated the next cycle of motion with the polishing tool, as shown in Figure 8e–h. The generated cracks in front of the abrasive grain in the first cycle could be improved, forming median cracks and lateral cracks on the subsurface. With the median cracks expanding, the SSD increased. The lateral cracks expanded in the direction of stress concentration, which could connect with the processed surface, leading to brittle removal. Figure 8 illustrates the initiation of cracks, expansion of cracks, and the final formation of median cracks during the VAP of SiC ceramics.

#### 3.3.2. Effect of the VAP vertical amplitude on the SSD

**Figure 9:**
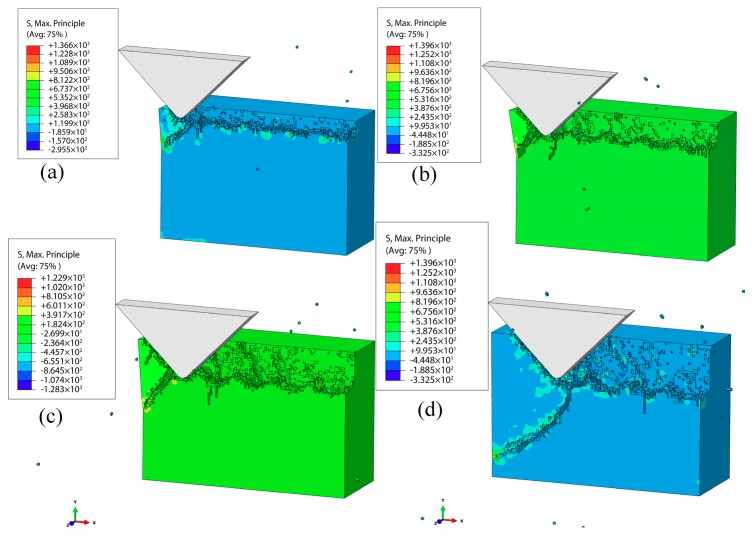
Effect of vertical amplitude on the SSD: (**a**) vertical amplitude is 1μm; (**b**) vertical amplitude is 2μm; (**c**) vertical amplitude is 3μm; (**d**) vertical amplitude is 4μm.

Figure 9a–d show the simulation results of the subsurface damages at VAP vertical amplitudes of 1, 2, 3, and 4 μm (the lateral amplitude was 1 μm, the frequency of vibration α was 30 kHz, the feed speed was 100 mm/s, and α = 90°), respectively. When the vertical amplitude was 1 μm (Figure 9a), the tiny median cracks could be observed at the end of the SiC workpiece. The cracks had an obvious expansion when vertical amplitude was increased to 2 μm, as shown in Figure 9b. Figure 9c,d show that the lengths of the cracks increased and the surface quality deteriorated as the vertical amplitude increased. The expansion of lateral cracks led to the brittle removal of SiC ceramics. According to the above simulation experiment, the surface damages and SSD increased when the vertical amplitude increased. As the vertical amplitude increased, the nominal indentation depth of the abrasive into the workpiece increased (the polishing force increased), which enhanced surface damages and SSD of the workpiece.

#### 3.3.3. Effect of the VAP lateral amplitude

**Figure 10:**
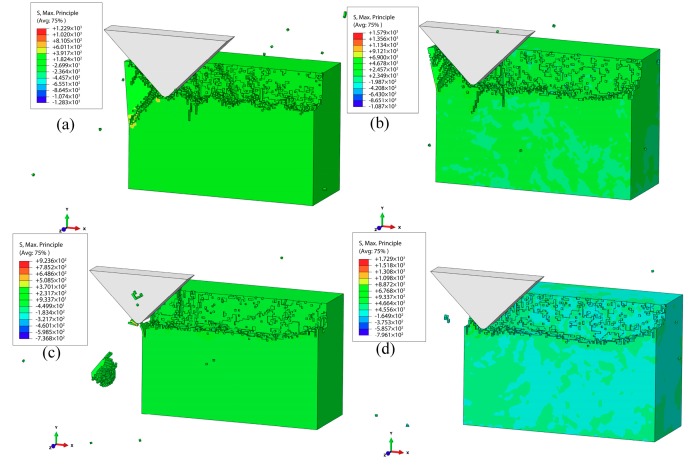
Effect of lateral amplitude on the SSD: (**a**) lateral amplitude is 1μm; (**b**) lateral amplitude is 3μm; (**c**) lateral amplitude is 4μm; (**d**) lateral amplitude is 5μm.

Figure 10a–d show the simulation results of the subsurface damages at VAP lateral amplitudes of 1, 3, 4, and 5 μm (the vertical amplitude was 3 μm, the frequency of vibration was 30 kHz, the feed speed was 100 mm/s, and α = 90°). Obviously, subsurface cracks and severely broken chips could be observed when the lateral amplitude was 1 μm. Meanwhile, the surface damage was also very serious. When the lateral amplitude was 2 μm, the subsurface cracks were reduced, and the surface and subsurface quality improved. There were almost no cracks initiated and the surface processed became smooth as the lateral amplitude increased to 5 μm. The surface integrity of the surface processed got better with the increase of the lateral amplitude. The surface residual stress of the workpiece decreased with the increase in abrasive movement range, which reduced or even eliminated the surface damages and SSD.

#### 3.3.4. Effect of the VAP Frequency on the SSD

**Figure 11:**
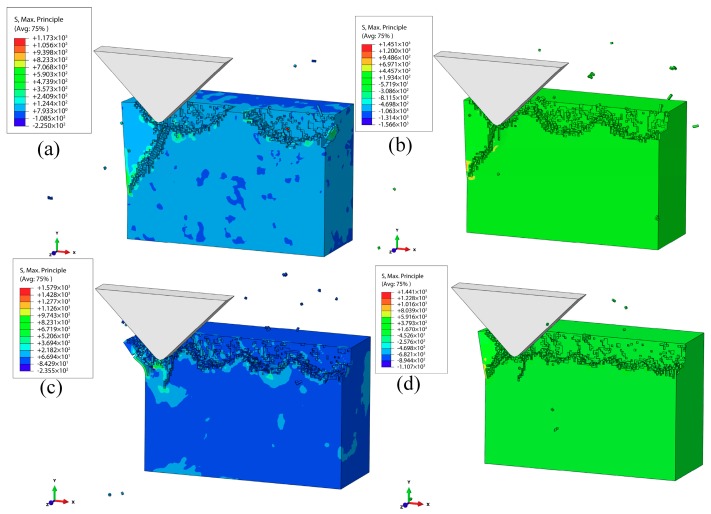
Effect of frequency on the SSD: (**a**) *f* = 10 KHz; (**b**) *f* = 15 KHz; (**c**) *f* = 20 KHz; (**d**) *f* = 30 KHz.

Figure 11 illustrates the simulation results of the subsurface damages for the machined workpiece at different VAP frequencies (10, 15, 20, and 30 kHz) when the vertical amplitude was 2 μm, the lateral amplitude was 1 μm, the speed was 100 mm/s, and the apex angle of abrasive was $\alpha =90^{{}^{\circ}}$ when the vibration frequency *f* was 10 kHz. Meanwhile, the surface quality of the workpiece was poor. When the VAP frequency increased to 30 kHz, the damages of the workpiece were reduced, as well as the SSD. Generally speaking, a relatively higher VAP frequency can decrease the SSD, thereby improving the quality of the subsurface. Thanks to the frequency increase, the abrasive particles were separated from the workpiece more often during the same processing time, causing the VAP force to decrease, which reduced the surface damages and SSD of the workpiece.

#### 3.3.5. Effect of the apex angle of abrasive on the SSD 

**Figure 12:**
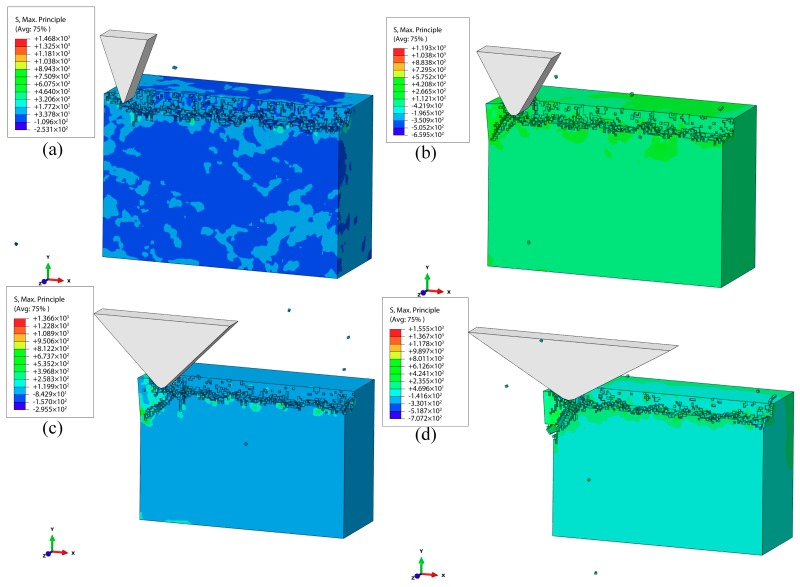
Effect of the apex angle of abrasive on the SSD: (**a**) α = 30°; (**b**) α = 60°; (**c**) α = 90°; (**d**) α = 30°.

Figure 12 illustrates the simulation results of the subsurface damages of the workpiece machined at various apex angles of abrasive α = 30–120° when the vertical amplitude was 1 μm, the lateral amplitude was 1 μm, the vibration frequency was 30 kHz, and the speed 100 mm/s. It was found that there were few tiny subsurface microcracks initiated and the polishing surface was relatively smooth when the apex angle of abrasive was 30°. Through the comparison with the results at other angles (60°, 90°, and 120°), it can be concluded that the SSD depth increased with the abrasive apex angle increasing. A decrease in the abrasive apex angle caused the interaction area between the workpiece and the abrasive to decrease, which could lead to a reduction in the residual stress of the processed surface. Therefore, selecting a small apex angle of abrasive (small abrasive size) is an effective method to reduce SSD depth.

## 4. Experiment

### 4.1. Experiment set-up

**Figure 13:**
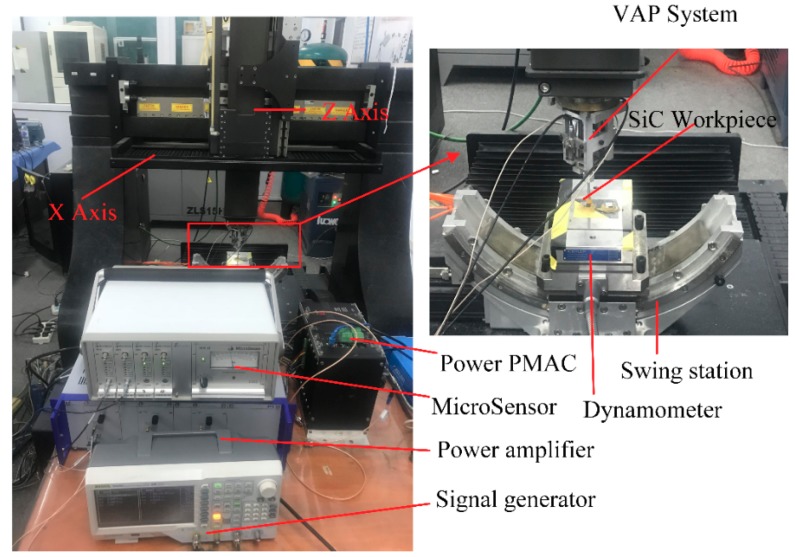
VAP experiment set-up.

**Figure 14:**
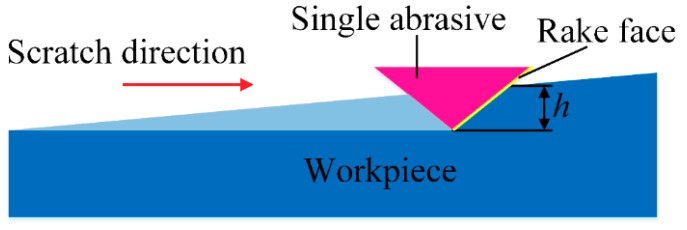
Scratch experiment set-up.

To explore the critical depth of SiC ceramics in the ductile–brittle transition, scratch tests were conducted using an independent precision polishing machine. The device could be controlled flexibly to determine the experimental parameters. The experiment set-up is shown in Figure 13. The VAP device was installed on the *Z*-axis, where the indenter was clamped in the tool holder. A 3D dynamometer (Kistler 9257B Switzerland) was used to control the force of the polishing tool on the workpiece surface. The SiC ceramic workpiece was rectangular with a size of 10 mm×10 mm×5 mm, which was bound to the dynamometer with bonding wax. The workpiece was ground with the abrasive slurry so that a smooth surface could be obtained to clearly observe the scratches. The diamond indenter was plunged into the sample, while the cutting speed was set to 0.3 mm/s and an inclined slope was set to 10 μm, as shown in Figure 14. In the vibration-assisted scratch tests, the voltages were applied to the PZT at different amplitudes, and the constant phase difference was $\varphi =90^{\circ }$. The machine parameters of the scratch tests are listed in Table 4. Then, the indenter was replaced by a polyurethane polishing tool to carry out the VAP experiment, in which the depth of cut increased continuously in the *Z*-direction. The machine parameters of the VAP single-factor tests are listed in Table 5. 

The surface roughness of the SiC sample was detected by the non-contact white-light interferometer (ZYGO, The NewView 8000). In order to study the depth of SSD on different parameters, a scanning electron microscope (SEM) was used to observe subsurface cracks. The cross-section polishing method was used to observe the subsurface of SiC ceramics. As the cracks were too tiny, the cross-sections of SiC ceramic samples should be polished with an argon ion polishing machine and chemically etched using HF solution to enable the visibility of subsurface cracks. Before being observed, the SiC sample should be ultrasonically cleaned several times. Finally, the maximum depth of cracks was selected from the micrographs.

**Figure 15:**
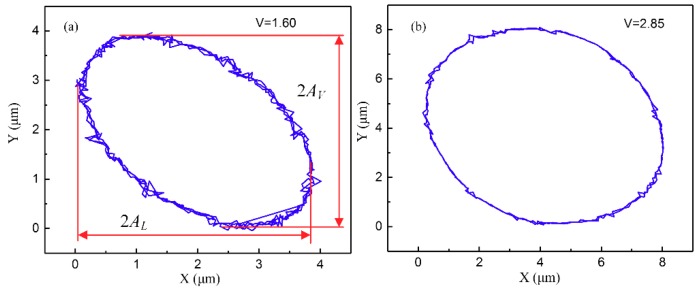
Tool trajectory of two-dimensional (2D) motions: (**a**) V = 1.60; (**b**) V = 2.85.

The actual 2D motions of the VAP device were measured with two displacement sensors, and then data were fitted by MATLAB. The typical results at different amplitudes of voltage are shown in Figure 15. Obviously, the lateral amplitude $A_{L}$ and the vertical amplitude $A_{V}$ increased with the applied voltage raised, and the values of $A_{L}$ and $A_{V}$ were approximately 1.9 μm and 2 μm at V = 1.6 V and V = 2.85 V, respectively. 

### 4.2. Experimental Results and Discussion

#### 4.2.1. Microscopic examination of the grooves with SEM

**Figure 16:**
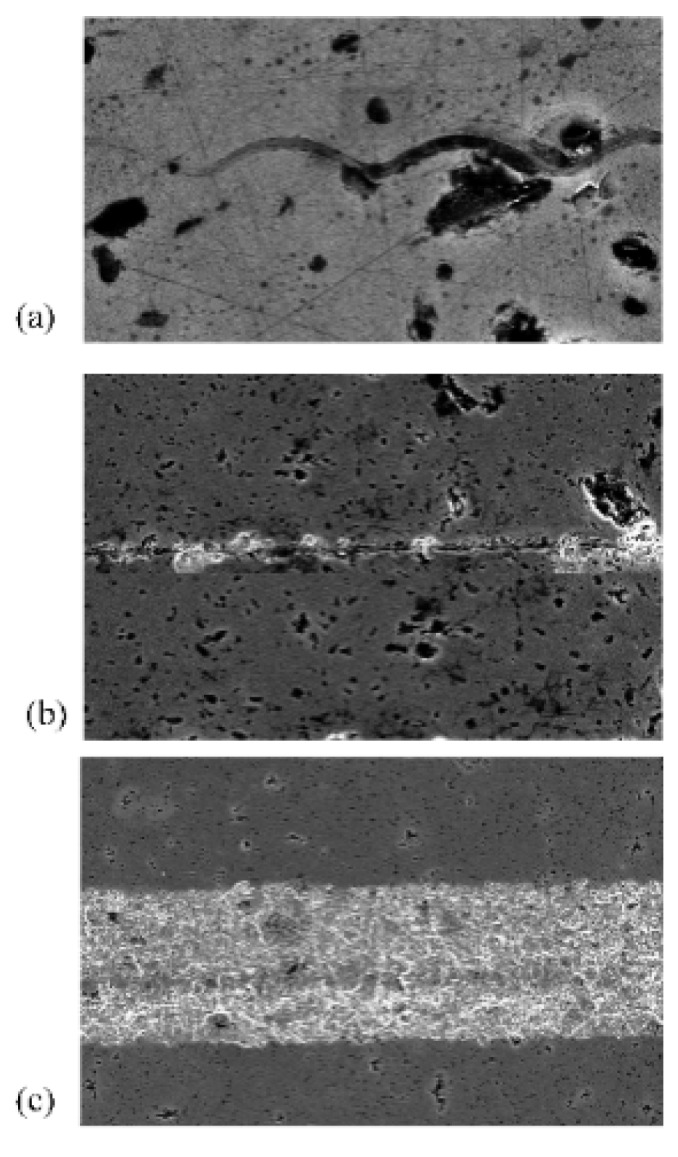
SEM images of the scratches on the SiC surface: (**a**) ductile removal; (**b**) brittle-to-ductile removal; (**c**) brittle removal.

Figure 16a–c show the SEM images of the scratches on the SiC sample surface, from which the surface damage feature of SiC ceramics can be observed. There were three removal forms: ductile removal, brittle-to-ductile transition, and brittle removal. In the initial state of the scratch (Figure 16a), ductile removal was the major form of removal with few chips and microcracks generated, and the surface morphology was smooth. As the scratch depth increased, obviously broken chips appeared along the direction of scratch, and some microcracks were generated near the grooves, as shown in Figure 16b. The microcracks were a sign of the brittle-to-ductile transition stage [41]. Figure 16c shows the brittle removal stage where severe damages (fracture removal) were generated near the grooves. Meanwhile, the surface quality was seriously deteriorated when the scratch depth continued to increase. To ensure processing of better surface, the VAP depth should be strictly controlled to obtain a workpiece with good surface integrity.

#### 4.2.2. Microscopic topography of the grooves

**Figure 17:**
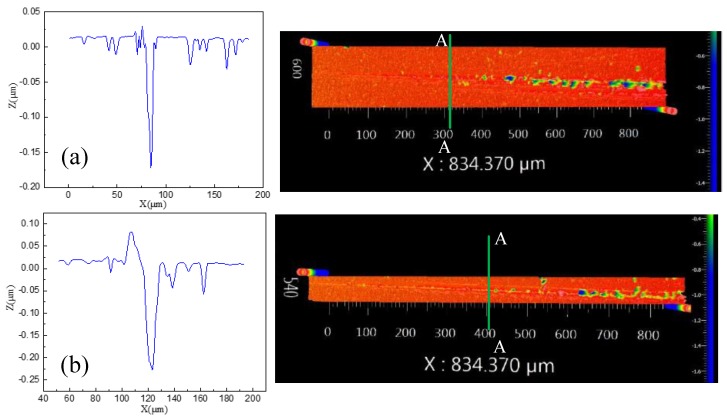
Micro-groove topography: (**a**) conventional scratch; (**b**) vibration scratch.

In order to investigate the variations of brittle–ductile transition in the scratch tests under different conditions, a white-light interferometer (ZYGO) could be used to obtain accurate depth values. Figure 17 shows the results of the tests. It is obvious that the scratched surface had a great deal of cracks and broken areas when the cut depth exceeded the critical value, indicating that brittle removal occurred. Figure 17a,b show the critical values of depth at the cross-section A–A region. It can be seen that the critical depth of cut at the vibration-assisted scratch was larger than in the conventional scratch test, and the critical cut depth increased with the lateral amplitude and the frequency. The values of critical cut depth are listed in Table 6. This effectively demonstrated that VAM can increase the critical depth of SiC. Using VAM technology is an effective method to reduce SSD.

#### 4.2.3. VAP experiments

**Figure 18:**
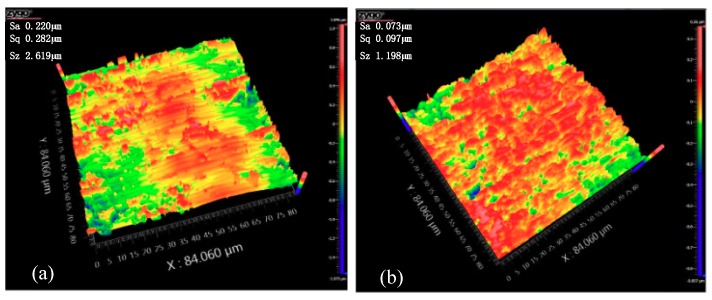
Three-dimensional (3D) surface topography: (**a**) conventional polishing surface; (**b**) VAP surface.

Based on the scratch tests, we can clearly conclude that VAM could increase the critical depth of cut. However, it is difficult to ensure processing in the ductile region due to the machine tool accuracy and workpiece assembly error. We could use the VAP method to reduce surface and subsurface damages. According to Table 5, a series of polishing experiments were conducted. The surface roughness of conventional polishing was 220 nm (Figure 18a), and the VAP experiment could reach a value of 73 nm (Figure 18b). The result indicated that VAP can improve the surface quality of SiC ceramics.

**Figure 19:**
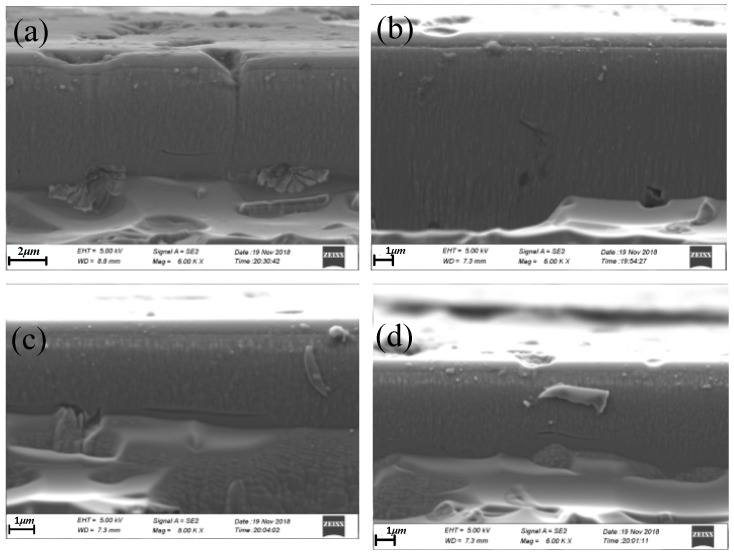
SEM images of SSD morphology of SiC: (**a**) median crack near the surface; (**b**) inclined crack of subsurface; (**c**) long lateral crack; (**d**) weak lateral crack.

Figure 19a–d show the images of subsurface cracks clearly observed beneath the VAP surface using SEM. During the VAP SiC ceramic process, a damage zone with microcracks was produced in the contact part between the abrasive grains and the workpiece due to the dislocation of the SiC crystal. Two types of SSD, including the lateral cracks of shell morphology and median cracks induced by polishing, can be clearly observed in the photograph. In Figure 19b, the direction of microcracks could be random due to the SiC ceramics involved in the Si and SiC phase, leading to the surface stress of workpiece being uneven. In general, propagation of the subsurface microcracks was initiated from the surface of the workpiece. As shown in Figure 19a, the median cracks were initiated due to the surface damage. Subsurface microcracks were distributed throughout the subsurface deformation layer. Because the interior of the material was not absolutely uniform, the crack extended to deeper locations where the microcracks were scattered at some relatively weak points of the SiC ceramics. In addition, it should be noted that the broken defects were also far away from the surface of the SiC ceramics in Figure 19b. These broken defects indicated that the SiC ceramics had certain defects with stomata and pits in the manufacturing process.

**Figure 20:**
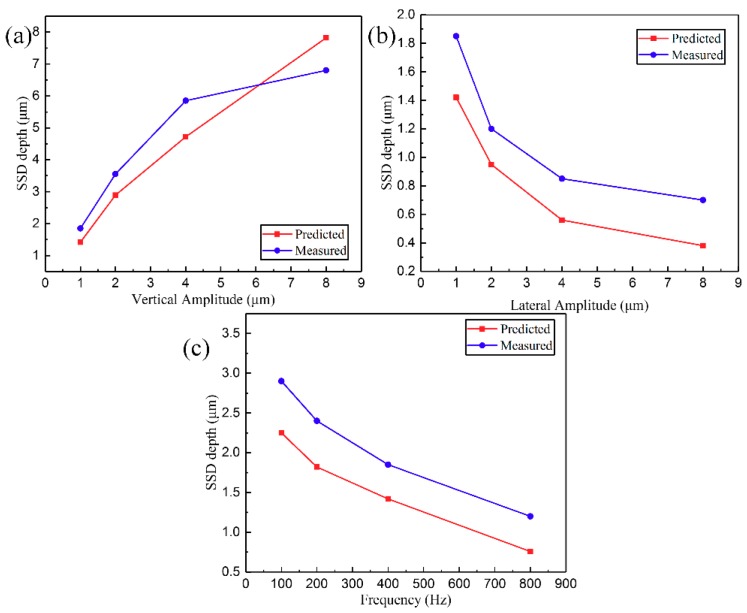
SSD depth as a function of different VAP parameters: (**a**) vertical amplitude; (**b**) lateral amplitude; (**c**) frequency.

Upon substituting the experimental parameters into Equation (22), the theoretically predicted value of SSD depth could be obtained. To reduce measurement time and cost, a 500 μm × 10μm area in the cross-section of SiC ceramics was polished on an argon ion polishing machine. Following the use of SEM to observe the SSD depth of SiC ceramics, the predicted and measured values are listed in Table 7. Figure 20 demonstrates the variations of the SSD with VAP vertical amplitude, lateral amplitude, frequency, and the apex angle of abrasive that included predicted and measured values. The SSD depth was positively correlated with the vertical amplitude of VAP because of the increase in abrasive extrusion depth and polishing force. On the other hand, increasing the amplitude and frequency of polishing decreased the normal force of the single abrasive, which gave rise to the formation of debris, and the decrease in plastic deformation of the surface layer and the SSD depth. Using a smaller abrasive can reduce material removal rate and polishing force, which leads to a decrease in the SSD depth.

According to the results and analysis, it is clearly shown that there is a good agreement between theoretical calculations and experiment with respect to the depth of SSD as a function of VAP parameters. However, there is a certain difference between theoretical values and experimental values. The main reasons are as follows: (1) the establishment of the mathematical model does not take into account some factors such as heat change, multi-abrasive coupling, tool wear, and so on; (2) elastic and plastic deformation of SiC ceramics is not considered in the theoretical equations; (3) the chemical reaction caused by etching is uncontrollable; (4) a smaller observation range results in the maximum SSD depth not being seen.

## 5. Conclusions

This study was carried out to investigate the critical cut depth in the vibration-assisted processing of SiC ceramics using a single diamond abrasive grain scratch model. First of all, the SSD of the SiC workpiece was analyzed based on the effects of different polishing parameters. Furthermore, combining the kinematic characteristics of VAP and the damage characteristics of the brittle materials, surface damages and SSD of SiC ceramics were analyzed. Finally, a theoretical model of SSD was established to predict the SSD values, and a 3D FEM simulation was used for qualitative analysis of the established model. The SSD value of the SiC workpiece was measured using the section polishing method, and the obtained results indicate the following:

(1) The critical cut depth of VAP is much larger than in conventional polishing in the scratch tests, indicating that VAP can increase the critical cut depth of SiC ceramics, and VAP is a superior method to improve the surface integrity of SiC ceramics in polishing.

(2) In the simulation, the removal process of SiC ceramics can be understood for the generation and propagation of microcracks. The simulation results indicate that the vertical amplitude (nominal VAP depth), the lateral amplitude, the frequency, and the abrasive grain size have the most significant effects on SSD. The SSD depth increases as the vertical amplitude and abrasive grain size increase. However, increasing the lateral amplitude and frequency can lead to a reduction of the SSD depth.

(3) The experiments were also carried out to analyze the effects of different processing parameters on the SSD depth and surface quality. In addition, the experimental results verified the validity of the theoretical and simulation results.

## Figures and Tables

**Table 1:** The material characteristic parameters of silicon carbide (SiC) ceramics.

Material Parameters	Value
Density *ρ* (kg/m^3^)	3215
Elastic modulus E (GPa)	454
Poisson’s ratio	0.25
Yield strength *σ* (MPa)	620
Specific heat (J/(kg·K))	526.3
Conductivity (W/(m·K))	180
Hardness (GPa)	29.4

**Table 2:** The model constants of SiC ceramics.

**Constitutive model**	$\rho_{0}\left(kg/m^{3}\right)$	G(GPa)	**A**	**N**	**B**	**M**	**C**
	3215	193	0.96	0.65	0.35	1.0	0.009
	$\sigma_{\max}^{i}\left(GPa\right)$	$\sigma_{\max}^{j}\left(GPa\right)$	HEL(GPa)	$P_{HEL}\left(GPa\right)$	β	${\overset{\cdot }{\varepsilon }}_{0}$	T(GPa)
	12.2	1.3	11.7	5.13	1.0	1.0	0.75
	β	$K_{1}\left(GPa\right)$	$K_{2}\left(GPa\right)$	$K_{3}\left(GPa\right)$			
	1.0	220	361	0			
Failure model	$D_{1}$	$D_{2}$	$\varepsilon_{f,\max}^{-pl}$	$\varepsilon_{f,\min}^{-pl}$	FS	Damage	
	0.48	0.48	1.2	0.0	0.2	0	

**Table 3:** Parameters for finite element model (FEM). VAP—vibration-assisted polishing.

VAP Parameters	Value
Vertical amplitude (μm)	1, 2, 3, 4
Lateral amplitude (μm)	1, 3, 4, 5
Frequencies (KHz)	10, 15, 20, 30
Abrasive angle (°)	30, 60, 90, 120

**Table 4:** Scratch test conditions.

Scratch Parameters	Conventional Scratch	Vibration-Assisted Scratch
Feed rate (mm/s)	0.3	0.3
Slope (μm)	10	10
Scratch distance (mm)	10	10
Scratch atmosphere	Dry	Dry

**Table 5:** VAP test conditions.

Group No.	Vertical Amplitude (μm)	Lateral Amplitude (μm)	Frequency (Hz)	Abrasive Size (μm)
1	1	2	400	W1
2	2	2	400	W1
3	4	2	400	W1
4	8	2	400	W1
5	1	1	400	W1
6	1	4	400	W1
7	1	8	400	W1
8	1	2	100	W1
9	1	2	200	W1
10	1	2	800	W1
11	1	2	400	W0.5
12	1	2	400	W1.5
13	1	2	400	W3

**Table 6:** The critical depth of cut at different parameters.

Grooving Test	Critical Depth of Cut (μm)
Conventional scratch	0.17
Vibration scratch (V = V, f = 100 Hz)	0.24

**Table 7:** The predicted and measured values of maximum subsurface damage (SSD) depth.

Group No.	Predicted SSD (μm)	Measured SSD (μm)
1	1.42	1.85
2	2.89	3.55
3	4.72	5.85
4	7.82	6.80
5	0.95	1.20
6	0.56	0.85
7	0.38	0.70
8	2.25	2.90
9	1.82	2.40
10	0.66	1.20
